# Factors Associated With Suicidal Ideation in College Students of Health Sciences

**DOI:** 10.1155/da/4397417

**Published:** 2025-07-28

**Authors:** Lourdes Luceño-Moreno, Daniel Vázquez-Estévez, Jesús Martín-García, Beatriz Talavera-Velasco

**Affiliations:** ^1^Department of Social and Work Psychology and Individual Differences, Universidad Complutense de Madrid Facultad de Psicología, Pozuelo de Alarcón, Community of Madrid, Spain; ^2^Department of Psychobiology and Methodology in Behavioral Sciences, Universidad Complutense de Madrid Facultad de Psicología, Pozuelo de Alarcón, Community of Madrid, Spain

**Keywords:** anxiety, college students, depression, health sciences, self-esteem, suicidal ideation

## Abstract

Suicide is the leading cause of death in people between 15 and 29 years of age, and its increase is worrying. Compared to other disciplines, university students of Health Sciences present a higher risk of suicidal ideation. The aim of this research was to identify which factors are associated with suicidal ideation in these students. A total of 412 university students from different Health Sciences specialties participated. The results indicate that higher levels of self-esteem, resilience, and perceived efficacy are associated with less suicidal ideation, while presenting more exhaustion, cynicism, stress, anxiety, and depression is related to a higher frequency of suicidal thoughts. Not seeking information about suicide and being in the first or second year were associated with more suicidal ideation. Not consuming alcohol or having close experiences of suicide was associated with less suicidal ideation. The profile of the university student with more suicidal ideation is one with high scores in depression and anxiety, and low scores in self-esteem. It is expected that these data can be considered in future suicide prevention programs.

## 1. Introduction

In the academic context, it has been shown that college students of Health Sciences present a higher risk of suicidal ideation [1, 2]. This is because, compared to students in other fields, Health Sciences students, especially medical students, tend to have a busy schedule and strict requirements in their clinical practice and are exposed to traumatic situations, such as the death of patients [1, 3]. Suicidal ideation is considered a predecessor variable to attempted suicide [4] and refers to thoughts related to harming oneself and wishing to take one's own life to solve problems, to being tired of living and feeling that one's life is worthless [5]. Some studies have detected a prevalence of 8.8% [6], while other authors have reported a prevalence of 19% in medical, dental, and pharmacy students [7].

Different risk factors associated with suicidal ideation in university students have been identified in research. They include feeling overloaded, having a psychiatric history, perceiving oneself as incapable of dealing with complex situations [8], the presence of depressive symptoms [9], nonbinary gender, taking antidepressants for more than 2 months, not perceiving good health, having anxiety symptoms [10] and having a family history of suicide [11]. Moreover, as the number of these factors increases, the probability of suicidal ideation increases [9]. On the other hand, alcohol consumption, stress, and test anxiety have also been identified as risk factors associated with suicidal thoughts [12, 13]. Among the various studies on anxious and depressive symptomatology in university students, it has been shown that in medical students the prevalence rate of anxiety symptoms is 33.8% [14]. In turn, 9.7% of university students simultaneously show symptoms of anxiety, depression, and stress [15]. On the other hand, having high levels of emotional exhaustion (burnout dimension) is related to suicidal ideation [16]. Burnout syndrome is characterized by physical, mental, or emotional exhaustion together with decreased motivation, lower performance, and the presence of negative attitudes towards oneself and others. Dabbagh et al. [17] analyzed different studies in their systematic review on burnout in medical students and found that the percentage of undergraduates suffering from burnout was between 13.4% and 67.1%.

Despite the above, it should be noted that there are other factors associated with a lower frequency of suicidal thoughts in college students, mainly self-esteem and resilience. Regarding self-esteem, this refers to the positive or negative attitude towards oneself and the evaluation of one's thoughts and feelings, in general, in relation to oneself [18]. Self-esteem has been identified as a predictor variable of suicidal ideation in college students [19]. Resilience refers to the capacity of people to recover and adapt to stressful situations [20]. It has been negatively associated with suicidal ideation in college students [21]. Finally, it is worth mentioning that obtaining adequate knowledge about suicide and the correct dissemination of this topic through the media can help prevent suicidal ideation and behavior [22].

In view of the above, this study aims to evaluate suicidal ideation in university students of Health Sciences. Specifically, the aim is to analyze the association between the factors studied (demographic variables, degree and year, knowledge of suicidal behavior, close experiences of suicidal behavior, alcohol consumption, anxiety, depression, self-esteem, resilience, burnout, and stress) and suicidal ideation. The main hypotheses of the study are as follows: (1) students who have less information about suicide or consume alcohol will have higher levels of suicidal ideation; (2) resilience will be associated with lower frequency of suicidal thoughts; (3) presenting high self-esteem will be associated with less suicidal ideation; (4) higher levels of burnout will be related to more suicidal ideation; (5) students with high scores in stress, anxiety, or depression will show more suicidal ideation.

## 2. Materials and Methods

### 2.1. Participants

A total of 412 university students of Health Sciences participated in this study, who were selected by nonprobability sampling. The sample consisted of 81 males (19.7%) and 331 females (80.3%), between 17 and 40 years of age, with a mean age of 21 (SD = 1.85). The most popular degree was psychology, with 231 students (56.1%), followed by nursing with 53 students (12.9%). The remaining degrees were medicine (8.7%), physiotherapy (3.1%), logopedics (7.1%), pharmacy (3.9%), nutrition and dietetics (1%), dentistry (2.9%), podiatry (0.5%), occupational therapy (1.4%), and double degree in psychology and logopedics (2.4%).

### 2.2. Variables and Instruments

An ad hoc questionnaire was conducted to assess the following variables: demographic variables (such as gender and age), degree and year, knowledge about suicidal behavior, close experiences of suicidal behavior, and alcohol consumption by asking “in the last month, how often have you consumed three alcoholic drinks in the same day?”.

Suicidal ideation: The Frequency of Suicidal Ideation Inventory (FSII) was applied [23]. This is a five items scale that assesses the frequency of suicidal ideation in the last 12 months in the nonclinical adult population. The scores for each item range from 1 (never) to 5 (almost every day). The total score ranges from 5 to 25, with higher scores indicating a higher frequency of suicidal ideation. This scale was validated in a sample of the general Spanish population with a minimum age of 18 years and shows adequate internal consistency (*α* = 0.89) and fit to a single factor [23]. In this study, the internal consistency was 0.90.

Self-esteem: The Spanish adaptation of the Rosenberg Self-esteem Scale [18, 24, 25] was applied. It is composed of 10 items focused on feelings of self-respect and self-acceptance. High scores indicate higher self-esteem. This scale shows high internal consistency in (*α* = 0.84) and an adequate fit to one factor in the Spanish validation with university students [24]. In the present research, Cronbach's alpha value was 0.89.

Stress: Spanish adaptation of the perceived stress scale—EPP [26, 27]. Composed of 14 items, it evaluates the extent to which people perceive that they have control over the demands of the environment. The higher the score, the greater the perceived stress. The Spanish adaptation in university students has adequate reliability (*α* = 0.73) and fit to a single factor [27]. In the present study, the internal consistency was 0.87.

Burnout: Maslach Burnout Inventory Student Survey—MBI-SS [28]. It consists of 16 items distributed across three scales: exhaustion (five items), cynicism (four items) and professional efficacy (six items). High scores on exhaustion and cynicism and low scores on professional efficacy indicate the presence of burnout. Schaufeli et al. [28] analyzed the psychometric properties of the MBI-SS in a cross-national study using a sample of university students from Spain, Portugal, and France. This study shows Cronbach's alpha values between 0.74 and 0.80 for the exhaustion scale, between 0.79 and 0.86 for the cynicism scale and between 0.67 and 0.76 for the professional efficacy scale. Likewise, the three-factor model shows a good level of fit. In the present research Cronbach's alpha values were 0.85, 0.89, and 0.85 for the exhaustion, cynicism, and professional efficacy scales, respectively.

Anxiety and depression: The Spanish adaptation of the Hospital Anxiety and Depression Scale—HADS [29, 30] was used. It is composed of 14 items divided into two scales; anxious symptoms (seven items) and depressive symptoms (seven items). A higher score indicates greater anxiety and/or depression. The Spanish adaptation, which included university students and the adult population in its sample, presents adequate reliability values, for HADS-A (*α* = 0.77) and for HADS-D (*α* = 0.71), as well as adequate adjustment to two factors [29]. In the present research Cronbach's alpha values were 0.83 and 0.77 in the anxiety and depression scales.

Resilience: The Spanish adaptation of the Brief Resilience Scale (BRS) [31, 32] was administered, which assesses the resilience capacity to cope with stressful situations and consists of six items. A higher score indicates a higher degree of resilience to cope with adversities. The scale presents adjustment to a single factor and an internal consistency of 0.83 in the Spanish validation, which used a general population sample, with a minimum age of 20 years [31]. In the present study, the internal consistency was 0.84.

### 2.3. Procedure

First, approval was obtained from the Ethics Committee of the Complutense University of Madrid (ref. Pr_2019_038). The data were collected by applying an online questionnaire through Google Forms, which included the instruments mentioned above. Before starting to answer the questions, participants had to give informed consent to continue. The informed consent included the purpose of the study, contact with research, and information on data confidentiality, anonymity, and legal aspects on personal data protection. The completion time was approximately 20 min.

### 2.4. Data Analysis

Descriptive analyses were performed on demographic variables, degree and year, knowledge of suicidal behavior, close experiences of suicidal behavior, alcohol consumption, anxiety, depression, self-esteem, resilience, burnout, stress, and suicidal ideation. Linear regression equations were performed using enter method to analyze the association between each of the variables independently with suicidal ideation, using the value of *R*^2^ and the standardized β coefficient. The objective was to determine the impact of each of the variables evaluated in predicting suicidal ideation and, to achieve this, dummy variables were used. A Dummy variable is a dichotomous variable constructed from an original categorical variable. For its creation, each level of the categorical variable gives rise to a new variable coded with values 0 (does not belong to the category) and 1 (belongs to the category) [33]. Finally, linear regression models were performed to evaluate which variables were related to suicidal ideation (gender, type of studies, course, degree, marital status, cohabitation, suicide knowledge, alcohol consumption, close experience of suicidal behavior, suicidal ideation, self-esteem, exhaustion, cynicism, efficacy, resilience, stress, anxiety, and depression). The model was estimated by least squares, using the forward extraction method.

## 3. Results

### 3.1. Demographic Variables, Related to Degree, and Suicidal Ideation

The year the students are in, specifically, being in first or second grade, is positively and significantly related to suicidal ideation. Regarding their knowledge about suicidal behavior, “not seeking information about suicide” is negatively and significantly associated with suicidal ideation. Not having consumed alcohol in the last month or having done so only on weekends is negatively and significantly associated with suicidal ideation. Finally, not having had any close experience of suicidal behavior is negatively associated with suicidal ideation. In the case of a close relative, it is positively and significantly associated with suicidal ideation (Table 1).

### 3.2. Psychological Variables Related to Suicidal Ideation

The variables self-esteem, resilience, and efficacy are independently and negatively related to suicidal ideation. Exhaustion, cynicism, stress, anxiety, and depression are positively and significantly associated with suicidal ideation (Table 2).

### 3.3. Regression Models for Suicidal Ideation

As shown in Table 3, the suicidal ideation model was significant, explaining 40.3% of the variance (*F* (3407) = 91.6919, *p* < 0.001). The variables that are positively related to suicidal ideation are; depression and anxiety. In contrast, the variable self-esteem was negatively associated with suicidal ideation (Table 3).

## 4. Discussion

The aim of this study was to evaluate suicidal ideation in college students of Health Sciences. Specifically, we examined the relationship between suicidal ideation and certain demographic variables (such as gender and age), degree and year, close experiences of suicidal behavior, knowledge of suicidal behavior, alcohol consumption, anxiety, depression, self-esteem, resilience, burnout, and stress.

The main results indicate that the factors associated with suicidal ideation are being in the first or second year, having close experiences of suicidal behavior in family members, not seeking information about suicide, presenting exhaustion, cynicism, stress, or symptoms of anxiety and depression. Regarding the year, no agreement has been found in the scientific literature on the academic year that is associated with more suicidal ideation, since many studies do not take this variable into account. Some authors point out that higher years (specifically, being in the third year) are related to more suicidal ideation [9]. On the other hand, and in line with the results of this research, an increase in symptoms of anxiety, depression, and suicidal ideation has been identified in the first academic year [34]. This could be due to these students' concerns about managing the transition to college, sleep quality, their academic performance, difficulties managing relationships, financial stability, or postdegree plans [35]. Regarding close experiences of suicidal behavior, other studies likewise show that, if the relationship with the person who has developed suicidal behavior is of a familial type, the probability of students presenting suicidal ideation is higher [6]. In relation to the knowledge that students have about suicide, those who do not seek information about suicide on social networks or other media present more suicidal ideation. This result would confirm part of the first hypothesis elaborated in this study. Other authors have concluded that students who take courses on suicide reduce suicidal ideation compared to those who do not participate in the courses. In addition, young people who take suicide courses have more resources to help others who present suicidal ideation, talk more openly about the subject, and present better resources for coping [36]. Students with higher scores on the exhaustion and cynicism scales present more suicidal ideation. This relationship between increased exhaustion and cynicism and greater likelihood of suicidal ideation has also been found in another study [37]. It is necessary to mention that stress increases the probability of suicidal thoughts and that students with higher levels of stress are also more likely to present a higher risk of mental disorders [6, 38]. Similarly, students who present moderate or severe symptoms of anxiety or depression and have had previous diagnoses of mental health problems are 10 times more likely to have suicidal ideation [39]. The results found with respect to the dimensions of burnout, anxiety, and depression would confirm hypotheses four and five of this research.

In this study, the following factors were identified as being related to a lower frequency of suicidal thoughts; not consuming alcohol, resilience, self-esteem, and efficacy. Not having consumed alcohol in the last month or having alcohol only on weekends decreases the probability of suicidal ideation in these students. In contrast, other authors point out that alcohol consumption poses a risk for suicidal ideation [40]. This could be explained by the association of alcohol consumption with a lack of problem-solving skills, avoidant coping, and acting impulsively in the face of negative situations without considering the possible consequences [41]. Alcohol consumption in these students has been explained by different factors, such as being in highly stressful environments, competitiveness in the academic context, workload, sleep problems, and burnout [42]. The results of this study on alcohol could not fully confirm the information regarding alcohol consumption in the first hypothesis developed. An association has been found between not consuming alcohol or consuming alcohol only on weekends and less suicidal ideation, but there has been no relationship between higher consumption and more frequency of suicidal thoughts, so further research on this variable is needed. Likewise, having high levels of resilience, self-esteem, or efficacy are associated in this study with less suicidal ideation. Resilience has been related to the perception of efficacy in college students, showing itself to be a mediating variable between general self-efficacy and mental health [43]. In scientific literature, these variables have been commonly related to less suicidal ideation [21]. These results would confirm the second and third hypotheses.

Among the factors that predict suicidal ideation, self-esteem, anxiety, and depression were identified. Other research also points to the importance of self-esteem as a predictor of suicidal ideation [44]. In addition, suicidal ideation has also been found to predict a higher negative self-view [45], which may be indicative that both variables feedback on each other. These results suggest that the levels of self-esteem, anxiety, and depression in these students should be considered when preventing suicidal ideation. Contrary to what other studies point out, resilience has not been identified as a protective variable for suicidal ideation [44]. Neither gender nor age have shown a relationship with suicidal ideation in this study.

This research has some limitations. The data were collected within a specific population (Health Sciences students), so the results cannot be generalized to college students from other disciplines. Also, the sampling was not random, and the study design is cross-sectional. It would be interesting, as a line of future research, to conduct a longitudinal study to temporally evaluate the effect of each of the variables examined in this research. On the other hand, most of the students in the sample were women. This is because the percentage of women is higher in Health Sciences degrees. It would be interesting in future research to have designs that examine possible differences in suicidal ideation with respect to gender or distinct disciplines. Finally, another limitation worth mentioning is the possibility that among the sample participants there may be students who may have mental health problems or individuals who show a special interest in issues related to mental health and suicide.

## 5. Conclusion

This study shows a profile of Health Sciences students who present characteristics associated with more suicidal ideation, such as high scores in depression and anxiety, and low scores in self-esteem. The results found may be relevant for designing suicide prevention programs for Health Sciences students in universities, especially those aimed at first-year students to help them in the following years of their education. These programs should be oriented to increase their self-esteem and efficacy, and to reduce the presence of anxious and depressive symptomatology. In addition, the burnout of these students should be reduced, reducing exhaustion, and cynicism. On the other hand, teaching information search guidelines on suicidal ideation and its associated factors could help prevent the occurrence of suicidal thoughts in the future. This study stresses the importance of raising awareness among students that lower alcohol consumption may help to reduce the occurrence of possible suicidal thoughts.

## Figures and Tables

**Table 1:** Association between demographic, degree-related variables, and suicidal ideation (*n* = 412).

Variable	Descriptive	Suicidal ideation		
	*N* (%)	*R* ^2^	*B* (*β*)	CI 95%
Gender				
Man	81 (19.7)	−0.002	0.122 (0.014)	−0.726, 0.970
Woman	331 (80.3)	—	—	—
Age M (SD)	21,22 (1.84)	0.004	0.146 (0.078)	−0.036, 0.329
Type of studies				
Grade	392 (95.1)	−0.002	—	—
Double grade	20 (4.9)	—	0.370 (0.023)	−1.197, 1.938
Course		0.026*⁣*^*∗*^		
1° and 2°	67 (16.3)		1.064 (0.113)*⁣*^*∗*^	0.023, 2.105
3°	112 (27.2)		—	—
4° and 5°	233 (56.6)		−0.626 (−0.089)	−10.401, 0.149
Degree		0.003		
Psychology	231 (56.1)		—	—
Nursing	53 (12.9)		−0.010 (−0.001)	−1.049, 1.029
Medicine	36 (8.7)		−1.074 (−0.087)	−2.297, 0.148
Another	92 (22.3)		−0.539 (−0.065)	−1.380, 0.302
Marital status		−0.002		
No stable partner	199 (48.3)		—	—
With stable partner	213 (51.7)		0.148 (0.21)	−0.526, 0.823
Cohabitation		−0.002		
Family residence	299 (72.6)		—	—
No family residence	113 (27.4)		−0.061 (−0.008)	−0.816, 0.695
Suicide knowledge		0.014*⁣*^*∗*^		
Suicide in the degree program	203 (49.3)		—	—
No suicide agenda in the degree program	209 (50.7)		0.699 (0.101)	−0.047, 1.445
Attending suicide courses	53 (12.9)		−0.191 (−0.018)	−1.223, 0.840
No attending suicide courses	359 (87.1)		—	—
Search for suicide information	117 (43)		—	—
Do not seek suicide information	235 (57)		0.767 (0.109)*⁣*^*∗*^	−1.480, −0.054
Imparting knowledge at the institute	107 (26)		−0.469 (−0.059)	−1.269, 0.332
No imparting knowledge at the institute	305 (74)		—	—
Knowing information about suicide	280 (68)		—	—
Not knowing information about suicide	132 (32)		−0.519 (−0.070)	−1.358, 0.321
Alcohol consumption				
Never	94 (22.8)	0.018*⁣*^*∗*^	−1.332 (−0.161) *⁣*^*∗∗*^	−2.268, −0.396
Less than 4 days per month	118 (28.6)	—	—	—
Weekends	160 (38.8)	—	−1.17 (−0.164) *⁣*^*∗∗*^	−1.992, −0.349
3 or more days a week	40 (9.7)	—	−0.726 (−0.062)	−1.965, 0.512
Close experience of suicidal behavior				
Yes	217 (52.7)	0.014*⁣*^*∗*^	—	—
No	195 (47.3)	—	−0.879 (−0.126)*⁣*^*∗*^	−1.548, −0.210
Suicidal behavior in				
Friend	149 (36.2)	—	—	—
No friend	263 (63.8)	—	−0.615 (−0.085)	−1.31, 0.08
Family	68 (16.5)	0.019*⁣*^*∗*^	1.093 (0.117)*⁣*^*∗*^	0.194, 1.992
Nonfamily	344 (83.5)	—	—	—
Another	37 (9)	—	—	—
No other	375 (91)	—	−0.876 (−0.072)	−2.044, 0.291

*⁣* ^*∗*^ *p* < 0.05.

*⁣* ^*∗∗*^ *p* < 0.01.

*⁣* ^*∗∗∗*^ *p* < 0.001.

**Table 2:** Association between psychological variables and suicidal ideation (*n* = 412).

Variable	Suicidal ideation		
	*R* ^2^	*B* (*β*)	CI 95%
Self-esteem	0.241*⁣*^*∗∗∗*^	−0.279 (0.492)*⁣*^*∗∗∗*^	0.231, 0.327
Efficacy	0.147*⁣*^*∗∗∗*^	−0.203 (−0.386)*⁣*^*∗∗∗*^	−0.25,−0.156
Exhaustion	0.104*⁣*^*∗∗∗*^	0.172 (0.326)*⁣*^*∗∗∗*^	0.123, 0.22
Cynicism	0.079*⁣*^*∗∗∗*^	0.189 (0.285)*⁣*^*∗∗∗*^	0.127, 0.25
Resilience	0.102*⁣*^*∗∗∗*^	−1.255 (−0.332)*⁣*^*∗∗∗*^	−1.613, −0.897
Stress	0.242*⁣*^*∗∗∗*^	0.243 (0.494)*⁣*^*∗∗∗*^	0.202, 0.285
Anxiety	0.242*⁣*^*∗∗∗*^	0.404 (0.494)*⁣*^*∗∗∗*^	0.335, 0.473
Depression	0.328*⁣*^*∗∗∗*^	0.621 (0.574)*⁣*^*∗∗∗*^	0.535, 0.707

*⁣* ^*∗*^ *p* < 0.05.

*⁣* ^*∗∗*^ *p* < 0.01.

*⁣* ^*∗∗∗*^ *p* < 0.001.

**Table 3:** Regression model for suicidal ideation (*n* = 412).

Variable	Suicidal ideation	
	*B* (*β*)	CI 95%
Depression (HADS)	0.361 (0.056)*⁣*^*∗∗∗*^	0.250, 0.471
Self-esteem (Rosenberg self-esteem scale)	−0.134 (−0.026)*⁣*^*∗∗∗*^	0.083, 0.185
Anxiety (HADS)	0.159 (0.040)*⁣*^*∗∗∗*^	0.081, 0.237

*⁣* ^*∗∗∗*^ *p* < 0.001.

## Data Availability

The data that support the findings of this study are available from the corresponding author upon reasonable request.
